# Ivermectin inhibits the sporogony of *Plasmodium falciparum* in *Anopheles gambiae*

**DOI:** 10.1186/1475-2875-11-381

**Published:** 2012-11-21

**Authors:** Kevin C Kobylinski, Brian D Foy, Jason H Richardson

**Affiliations:** 1 Entomology Branch, Walter Reed Army Institute of Research, 503 Robert Grant Ave, Silver Spring, MD, 20910, USA; 2 Department of Microbiology, Immunology and Pathology, Arthropod-borne and Infectious Diseases Laboratory, Colorado State University, 1692 Campus Delivery, Fort Collins, CO, 80523-1692, USA

**Keywords:** *Anopheles gambiae*, *Plasmodium falciparum*, Ivermectin, Transmission

## Abstract

**Background:**

When ingested in a blood meal, ivermectin has been shown to reduce the survivorship of *Anopheles gambiae* in the laboratory and field. Furthermore, ivermectin mass drug administrations in Senegal have been shown to reduce the proportion of *Plasmodium falciparum*-sporozoite-containing *An. gambiae*. This study addresses whether ivermectin inhibits sporogony of *P. falciparum* in *An. gambiae.*

**Methods:**

*Anophele gambiae* s.s. G3 strain were fed two concentrations of ivermectin (LC_25_ and LC_5_) along with *P. falciparum* NF54 in human blood meals at staggered intervals. Mosquitoes ingested ivermectin concurrent with parasites (DPI 0), or at three (DPI 3), six (DPI 6), and nine (DPI 9) days post parasite ingestion, or three days prior (DPI −3) to parasite ingestion. Mosquitoes were dissected at seven, twelve or fourteen days post parasite ingestion and either oocyst or sporozoite prevalence was recorded. To determine if *P. falciparum* sporozoite-containing *An. gambiae* were more susceptible to ivermectin than uninfected controls, survivorship was recorded for mosquitoes which ingested *P. falciparum* or control blood meal on DPI 0 and then a second blood meal containing ivermectin (LC_25_) on DPI 14.

**Results:**

Ivermectin (LC_25_) co-ingested (DPI 0) with parasites reduced the proportion of *An. gambiae* that developed oocysts (*χ*^2^ = 15.4842, *P* = 0.0002) and sporozoites (*χ*^2^ = 19.9643, *P* < 0.0001). Ivermectin (LC_25_) ingested DPI 6 (*χ*^2^ = 8.5103, *P* = 0.0044) and 9 (*χ*^2^ = 14.7998, *P* < 0.0001) reduced the proportion of *An. gambiae* that developed sporozoites but not when ingested DPI 3 (*χ*^2^ = 0.0113, *P* = 1). Ivermectin (LC_5_) co-ingested (DPI 0) with parasites did not reduce the proportion of *An. gambiae* that developed oocysts (*χ*^2^ = 4.2518, *P* = 0.0577) or sporozoites (*χ*^2^ = 2.3636, *P* = 0.1540), however, when ingested DPI −3 the proportion of *An. gambiae* that developed sporozoites was reduced (*χ*^2^ = 8.4806, *P* = 0.0047). *Plasmodium falciparum* infection significantly reduced the survivorship of *An. gambiae* that ingested ivermectin (LC_25_) on DPI 14 compared to control mosquitoes that ingested a primary blood meal without parasites (*χ*^2^ = 4.97, *P* = 0.0257).

**Conclusions:**

Ivermectin at sub-lethal concentrations inhibits the sporogony of *P. falciparum* in *An. gambiae*. These findings support the utility of ivermectin for *P. falciparum* transmission control.

## Background

Novel *Anopheles* vector control methods are needed for current malaria elimination and eradication efforts [1]. Ivermectin mass drug administration (MDA) to humans has been suggested as a possible vector control method to reduce *Plasmodium* transmission [2-8]. Ivermectin MDA to humans meets several of the criteria outlined by the Malaria Eradication Research Agenda Consultative Group on Vector Control for novel vector control interventions, including: the ability to target exophagic and exophilic vectors, integration with current vector control efforts [5,6], novel mode of action [9,10] from the currently used insecticides [11], avoids known mosquito behavioural and physiological resistance mechanisms, and can affect the vector population age structure [7].

The vectorial capacity equation provides a framework that defines the factors that regulate *Plasmodium* transmission by *Anopheles* vectors [12]. The current vector control strategies of indoor residual spraying with insecticides and long lasting insecticide treated nets primarily affect the daily probability of mosquito survivorship and/or reduce vector-host contact [13,14]. However, there are several other variables in the vectorial capacity equation that vector control methods could impact which would reduce *Plasmodium* transmission.

The most influential variable of vectorial capacity is the daily probability of mosquito survivorship [12,15]. *In vitro* laboratory studies have demonstrated that ivermectin can reduce the daily probability of survivorship of numerous *Anopheles Plasmodium-*vectors, including *Anopheles gambiae* s.s. at physiologically relevant concentrations found in humans after ingestion of ivermectin [4,16,17]. The second most influential variable of the vectorial capacity equation is the daily probability of a mosquito feeding on a human, which is a factor of the mosquito host preference index and the feeding frequency. *Anopheles gambiae* s.s. blood-feeds almost exclusively on humans with a median host preference index of 0.939 [18]. The feeding frequency of *An. gambiae* s.s. was significantly delayed when ivermectin was ingested in a blood meal at mosquito sub-lethal concentrations [4,19]. The vector density relation to the host is another variable of vectorial capacity that may be affected by ivermectin MDA. Ivermectin not only reduces the survivorship of *An. gambiae* s.s., but it also causes a knockdown effect and a recovery delay post blood feeding [19] which likely further reduces mosquito populations due to desiccation, starvation, and predation. Ivermectin has also been shown to reduce *An. gambiae* fecundity [16]. This laboratory evidence and modelling data [7] suggests that vector density in relation to the host may be temporarily reduced after ivermectin MDA to humans, but this remains to be demonstrated in a field setting.

Ivermectin MDAs to humans are performed annually in many parts of sub-Saharan Africa for *Onchocerca volvulus* transmission suppression by the African Program for Onchocerciasis Control (APOC) and *Wuchereria bancrofti* transmission suppression by the Global Programme to Eliminate Lymphatic Filariasis [20]. In south-eastern Senegal, indoor-resting blood-fed *An. gambiae* s.l. were collected before and after routine APOC ivermectin MDAs from three pairs of treated and control villages and this work clearly demonstrated that *An. gambiae* s.s. survivorship was significantly reduced for approximately one week following ivermectin MDA [5]. Further analysis of field-collected mosquitoes revealed that ivermectin MDA also reduced the proportion of *Plasmodium falciparum* sporozoite-containing (*i.e.* infectious) *An. gambiae* s.s. for at least two weeks post-MDA [6].

There are several possible explanations for this reduction in the proportion of *P. falciparum*-infectious *An. gambiae* s.s, which are not exclusive of each other. Modelling has predicted that the *An. gambiae* population age structure following ivermectin MDA temporarily shifts to younger age classes, therefore, fewer *P. falciparum*-infectious mosquitoes are present for several weeks [7]. Field work is currently being performed in Senegal to gather empirical evidence as to whether this population age structure shift occurs. It may also be that *P. falciparum*-infectious mosquitoes are more susceptible than non-infected mosquitoes to ivermectin and thus are preferentially removed from the population of mosquitoes. Finally, ivermectin may be sporontocidal and thus lower the proportion or prevalence of *Anopheles* ingesting an infective *Plasmodium* blood meal that successfully become infectious or lengthen the extrinsic incubation period of *Plasmodium* in the *Anopheles* vector. These latter three possibilities were explored below in laboratory experiments with *P. falciparum* and *An. gambiae* s.s.

## Methods

### Parasites

Gametocyte cultures of *P. falciparum* (NF54) line were cultured *in vitro* following the modified Trager and Jensen method [21,22] in human erythrocytes group O+ (Key Biologics LLC, Memphis, TN) and RPMI 1640 medium (Life Technologies, Grand Island, NY) supplemented with 25 mM HEPES, 25 mM NaHCO_3_, 50 mg/L hypoxanthine (Sigma-Aldrich, St. Louis, MO) and 10% (v/v) type A+ human serum (Key Biologics LLC, Memphis, TN). Media was changed daily and parasites were blood fed to mosquitoes 15 days post subculture. For mosquito blood meals cultures were transferred from flasks to 50 ml conical tubes and centrifuged at 1600 RPMs for ten minutes at 37°C and culture media supernatant was removed. Red blood cells (RBCs) and human serum were mixed in a 1:1 ratio and fed to mosquitoes via glass membrane feeders sealed with parafilm at 37°C.

### Mosquitoes

*Anopheles gambiae* s.s. (G3 strain) were obtained from the National Institutes of Health, raised at 26 -27°C, 80% relative humidity, and a 12:12 light:dark cycle. Larvae were raised on a diet of ground Tetramin® fish food. Adults were provided 10% sucrose solution *ad libitum*. Adult mosquitoes were between four and seven days post emergence when they were fed a *P. falciparum-*infectious blood meal.

### LC_50_ calculations

The powdered ivermectin formulation was obtained from Sigma-Aldrich (St. Louis, MO). Ivermectin was diluted in dimethyl sulfoxide (DMSO) to 10 mg/ml and aliquots were frozen at −20°C. Frozen aliquots of ivermectin were thawed and serially diluted in phosphate buffered saline (PBS) prior to addition to blood meals consisting of packed RBCs and human serum mixed 1:1 heated to 37°C prior to mixing. Ten μl of varied concentrations of ivermectin in PBS were added to 990 μl of blood meal to reach the final concentrations offered to mosquitoes. Multiple concentrations of ivermectin were fed to adult *An. gambiae* s.s. between four and seven days post emergence to determine the lethal concentration that killed 50%, 35%, 25%, and 5% of the mosquitoes (LC_50_, LC_35_, LC_25_, and LC_5_). After blood feeding, 50 fully engorged mosquitoes were gently transferred by aspiration to 0.5 L cardboard cartons and held in a different incubator at 26°C, 80% relative humidity, and a 12:12 light:dark cycle. Engorged mosquitoes were held for seven days with access to 10% sucrose solution and survivorship was monitored every 24 hours and dead mosquitoes were removed from the containers daily. Six replicates were performed.

### Effect of ivermectin on *Plasmodium falciparum* sporogony

*Anopheles gambiae* s.s. were fed cultured gametocytaemic RBCs with various concentrations of ivermectin, LC_25_ and LC_5_, and at various time points in relation to the infectious blood meals. Ivermectin-(LC_25_)-containing blood meals were administered concomitantly with parasites (DPI 0), or three (DPI 3), six (DPI 6), and nine (DPI 9) days after (post) the infectious (DPI) blood meal. Ivermectin-(LC_5_)-containing blood meals were administered concomitantly with parasites (DPI 0), or three (DPI −3) days before parasites. Control mosquitoes were fed in a separate container with the same blood meal schedule, only ivermectin was replaced with DMSO at equivalent concentrations in PBS. After blood feeding, fully engorged mosquitoes were gently transferred by aspiration to new 3.8 L cardboard cartons and held in a different incubator at 26°C, 80% relative humidity, and a 12:12 light:dark cycle. Mosquitoes were held for fourteen days after the infectious blood feed with 10% sucrose solution *ad libitum*. Mosquitoes fed ivermectin (LC_25_ and LC_5_) concomitantly with infectious gametocytes (DPI 0) were dissected on day post infection (DDPI) 7 and oocysts were enumerated. Midguts were dissected with forceps into saline on a microscope slide and stained for 15 minutes in methylene blue (0.1%) (Fisher Scientific, Fair Lawn, NJ), pressed between a cover slip and viewed at 200X magnification with a MT5000 Series biological microscope (Meiji Techno, Santa Clara, CA). A delay in blood meal digestion caused by ivermectin [4] ingested on DPIs 3 and 6 obscured the ability to view oocysts on the midgut, therefore, prevalence and intensity were not determined at those time points. On DDPI 12 and 14, salivary glands were dissected with forceps into saline on a microscope slide, pressed between a cover slip, and viewed at 200X magnification with a MT5000 Series biological microscope. Prevalence of sporozoites in salivary glands were recorded for each mosquito. Four replicates of ivermectin (LC_5_ or LC_25_) concentration and DPI (−3, 0, 3, 6, or 9) combinations were performed and approximately twenty mosquitoes were dissected for each control and treatment group on each DDPI. Two additional replicates of ivermectin (LC_25_) DPI 0 were performed in order to obtain enough visible midguts with oocyst infections for statistical analyses.

### Effect of *Plasmodium falciparum* infection on mosquito survivorship post ivermectin ingestion

*Anopheles gambiae* s.s. were fed either gametocytaemic or non-gametocytaemic control blood meals and held in the insectary for fourteen days in 3.8 L cartons. On DPI 14, ten mosquitoes were dissected as described above to confirm at least 90% *P. falciparum* sporozoite presence in salivary glands. If the mosquitoes met the infection criteria status, then both infected and control cartons were fed a secondary ivermectin (LC_25_)-containing blood meal. Approximately 50 mosquitoes were transferred by aspiration to 0.5 L cartons and held as described above. Mosquito survivorship was monitored every 24 hours and dead mosquitoes were removed from the containers daily until all mosquitoes were dead. Five replicates were performed.

### Statistical analysis

A non-linear mixed model with probit analysis was used to calculate LC_50_, LC_35,_ LC_25_, and LC_5_ values as described previously [4]. The proportion of mosquitoes with oocysts or sporozoites was compared by Fisher’s Exact test. In order to determine if ivermectin lengthened the extrinsic incubation period the rates of change in the proportion of sporozoite positive mosquitoes between DDPI 12 and DDPI 14 were analyzed by logistic regression (SAS Proc GLIMMIX) [23] with effects for treatment (ivermectin, control), period (DDPI 12, DDPI 14), and treatment by period with replicate as a random effect. The treatment by period interaction tests whether the change in the proportion of sporozoite positive mosquitoes between DDPI 12 and DDPI 14 differs between ivermectin and control treatments. These above statistical analyses were performed with Statistical Analysis Software (SAS Institute Inc., Cary, NC). Oocyst intensity was compared by the Mann–Whitney *U* test. To determine if *P. falciparum* infection altered mosquito survivorship post-ivermectin ingestion survival analysis data were analysed by the Mantel-Cox method. Mann–Whitney U tests and Mantel-Cox tests were performed with PRISM 5 (GraphPad Software, Inc., San Diego, CA).

## Results

### LC_50_ calculations

The lethal concentrations of ivermectin fed to *An. gambiae* s.s. were calculated as: LC_50_ = 15.9 ng/ml 95% C.I. [14.6, 17.3], LC_35_ = 12.7 ng/ml [11.3, 13.9], LC_25_ = 10.7 ng/ml [9.3, 12.0], LC_5_ = 6.1 ng/ml [4.8, 7.4] (n = 3355).

### Effect of ivermectin on *Plasmodium falciparum* sporogony

**Figure 1:**
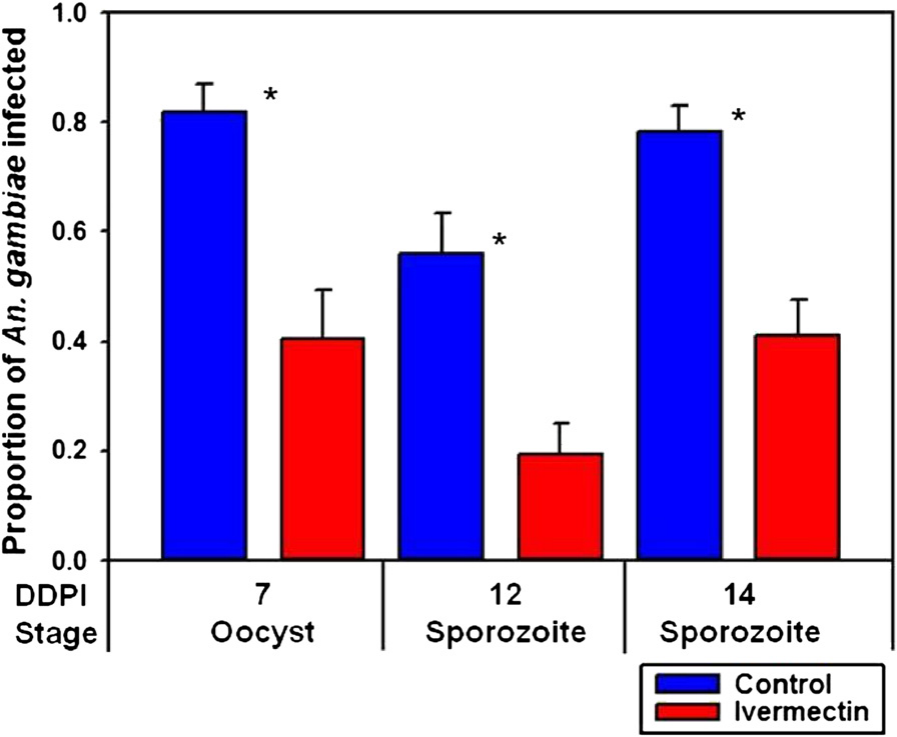
**Proportion of *Anopheles gambiae* that were infected with *Plasmodium falciparum* when ivermectin (LC**_**25**_**) was co-ingested with parasites (DPI 0).** When ivermectin (LC_25_) was co-ingested with *P. falciparum* at DPI 0 it reduced the prevalence or proportion of *An. gambiae* that developed oocysts at DDPI 7 (*χ*^2^ = 15.4842, *P* = 0.0002, n = 89) and sporozoites at DDPI 12 (*χ*^2^ = 13.4741, *P* = 0.0003, n = 97) and at DDPI 14 (*χ*^2^ = 19.9643, *P* < 0.0001, n = 136). * significantly different *P* < 0.05, DPI = days post infection, DDPI = dissected at day post infection.

**Figure 2:**
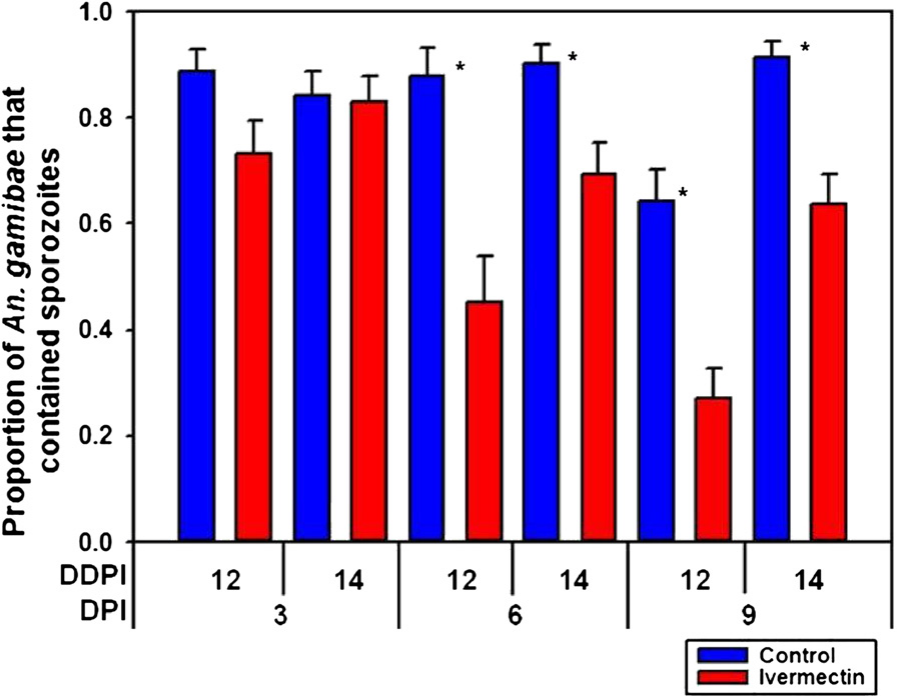
**Proportion of *Anopheles gambiae* that contained sporozoites when ivermectin (LC**_**25**_**) was ingested DPIs 3, 6, 9.** When ivermectin (LC_25_) was fed at DPI 3 post parasites it did not reduce the proportion of *An. gambiae* that contained sporozoites at DDPI 12 (*χ*^2^ = 4.3718, *P* = 0.0556, n = 112) or at DDPI 14 (*χ*^2^ = 0.0113, *P* = 1, n = 145). However, when ivermectin (LC_25_) was fed at DPIs 6 and 9 post parasites it reduced the proportion of *An. gambiae* that contained sporozoites on DPI 6 at DDPI 12 (*χ*^2^ = 12.4919, *P* = 0.0006, n = 95) and at DDPI 14 (*χ*^2^ = 8.5103, *P* = 0.0044, n = 135) and on DPI 9 at DDPI 12 (*χ*^2^ = 16.2056, *P* < 0.0001, n = 132) and at DDPI 14 (*χ*^2^ = 14.7998, *P* < 0.0001, n = 161). * significantly different *P* < 0.05, DPI = days post infection, DDPI = dissected at day post infection.

When ivermectin (LC_25_) was co-ingested with *P. falciparum* (ie. DPI 0) it reduced the prevalence or proportion of *An. gambiae* that developed oocysts at DDPI 7 (*χ*^2^ = 15.4842, *P* = 0.0002, n = 89) and sporozoites at DDPI 12 (*χ*^2^ = 13.4741, *P* = 0.0003, n = 97) and at DDPI 14 (*χ*^2^ = 19.9643, *P* < 0.0001, n = 136) (Figure 1). The oocyst intensity per successfully infected mosquito fed ivermectin (LC_25_) (mean = 21.0 ± 2.93 s.e.) at DPI 0 was not reduced relative to controls (mean = 21.5 ± 2.94 s.e.) (*P* = 0.8139, n = 136). When ivermectin (LC_25_) was ingested on DPI 3, it did not reduce the proportion of *An. gambiae* that contained sporozoites at DDPI 12 (*χ*^2^ = 4.3718, *P* = 0.0556, n = 112) or at DDPI 14 (*χ*^2^ = 0.0113, *P* = 1, n = 145) (Figure 2). However, ivermectin (LC_25_) ingested on DPI 6 and DPI 9 reduced the proportion of *An. gambiae* that contained sporozoites at DDPI 12 and DDPI 14 [DPI 6 at DDPI 12 (*χ*^2^ = 12.4919, *P* = 0.0006, n = 95) and DPI 6 at DDPI 14 (*χ*^2^ = 8.5103, *P* = 0.0044, n = 135); DPI 9 at DDPI 12 (*χ*^2^ = 16.2056, *P* < 0.0001, n = 132) and DPI 9 at DDPI 14 (*χ*^2^ = 14.7998, *P* < 0.0001, n = 161)] (Figure 2).

**Figure 3:**
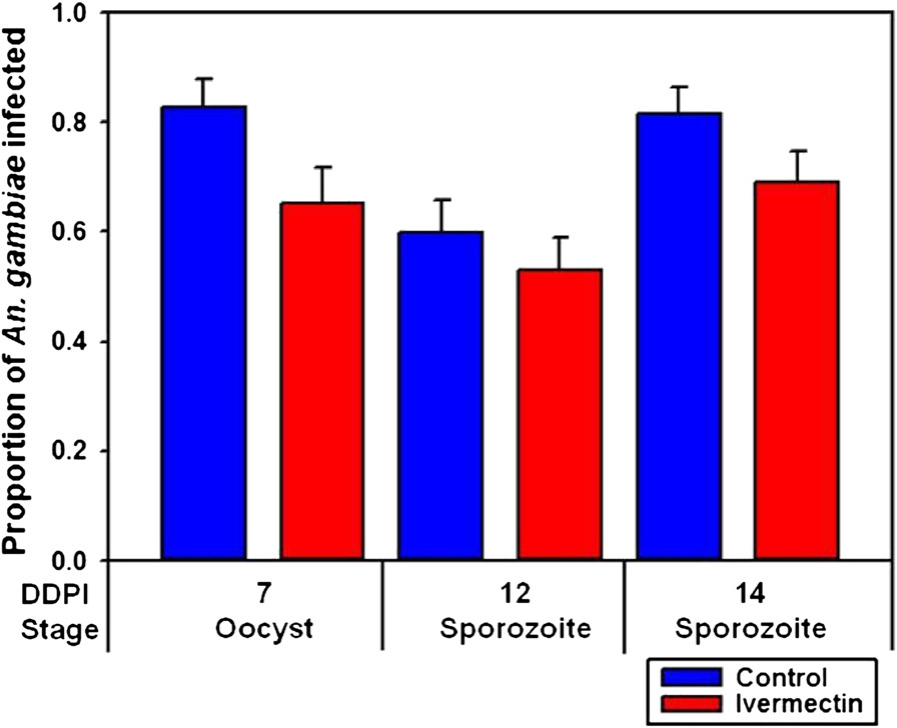
**Proportion of *Anopheles gambiae* that were infected with *Plasmodium falciparum* when ivermectin (LC**_**5**_**) was co-ingested with parasites (DPI 0).** When ivermectin (LC_5_) was co-ingested with *P. falciparum* on DPI 0 it did not reduce the proportion of *An. gambiae* that developed oocysts at DDPI 7 (*χ*^2^ = 4.2518, *P* = 0.0577, n = 115) or sporozoites at DDPI 12 (*χ*^2^ = 0.5937, *P* = 0.5101, n = 149) or at DDPI 14 (*χ*^2^ = 2.3636, *P* = 0.1540, n = 156).

**Figure 4:**
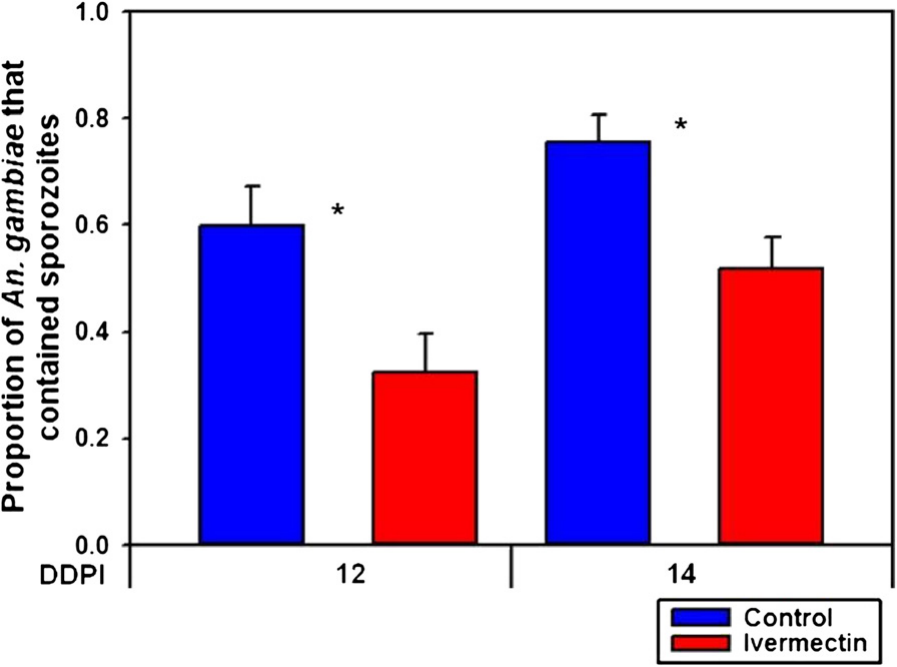
**Proportion of *Anopheles gambiae* that contained sporozoites when ivermectin (LC**_**5**_**) was ingested DPI −3.** When ivermectin (LC_5_) was ingested three days before parasites (DPI −3) it reduced the proportion of *An. gambiae* that contained sporozoites at DDPI 12 (*χ*^2^ = 4.6333, *P* = 0.0406, n = 95) and at DDPI 14 (*χ*^2^ = 8.4806, *P* = 0.0047, n = 155).

When ivermectin (LC_5_) was co-ingested with *P. falciparum* (DPI 0), it did not reduce the prevalence or proportion of *An. gambiae* that developed oocysts at DDPI 7 (*χ*^2^ = 4.2518, *P* = 0.0577, n = 115) or sporozoites at DDPI 12 (*χ*^2^ = 0.5937, *P* = 0.5101, n = 149) or at DDPI 14 (*χ*^2^ = 2.3636, *P* = 0.1540, n = 156) (Figure 3). The oocyst intensity per successfully infected mosquito fed ivermectin (LC_5_) on DPI 0 (mean = 15.4 ± 2.53 s.e.) was not reduced compared to controls (mean = 13.1 ± 2.34 s.e.) (*P* = 0.9278, n = 84). However, when ivermectin (LC_5_) was ingested three days before a *P. falciparum* blood meal (DPI −3) it reduced the proportion of *An. gambiae* that contained sporozoites at DDPI 12 (*χ*^2^ = 4.6333, *P* = 0.0406, n = 95) and at DDPI 14 (*χ*^2^ = 8.4806, *P* = 0.0047, n = 155) (Figure 4).

Ivermectin (LC_25_) did not delay the extrinsic incubation period of *P. falciparum* when ingested on DPI 0 (F-value = 0.01, *P* = 0.9308, n = 233), DPI 3 (F-value = 1.89, *P* = 0.2116, n = 257), DPI 6 (F-value = 0.27, *P* = 0.6182, n = 230), or DPI 9 (F-value = 0.08, *P* = 0.7796, n = 295) nor did ivermectin (LC_5_) delay sporogony when ingested at DPI 0 (F-value = 0.59, *P* = 0.4622, n = 304) or DPI −3 (F-value = 0.01, *P* = 0.9238, n = 250).

### Effect of *Plasmodium falciparum* infection on mosquito survivorship post ivermectin ingestion

**Figure 5:**
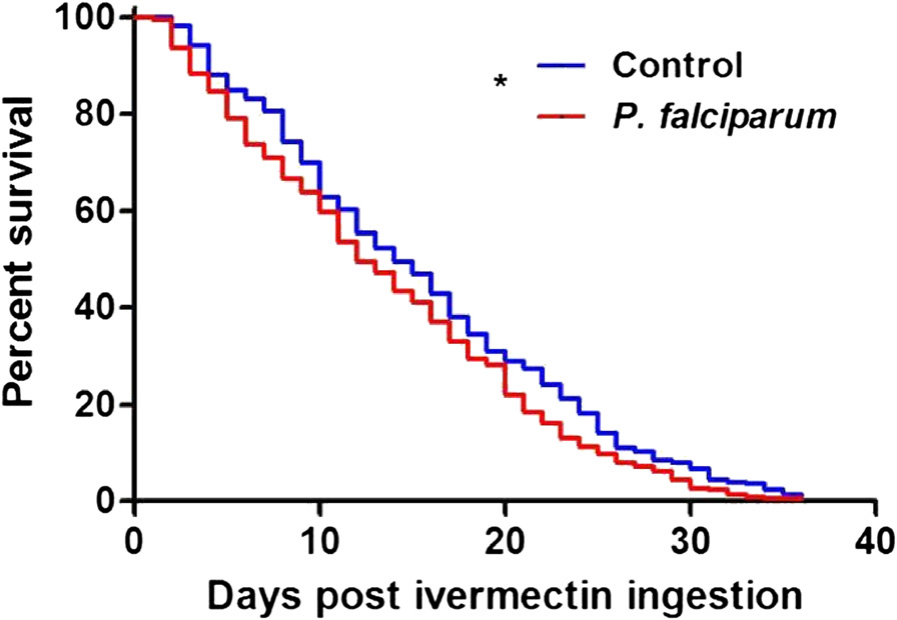
**Survivorship of *Anopheles gambiae* that ingested ivermectin (LC**_**25**_**) at DPI 14. ***Plasmodium falciparum* infection significantly reduced the survivorship of *An. gambiae* that ingested ivermectin on DPI 14 compared to control mosquitoes that received a primary blood meal without parasites (*χ*^2^ = 4.97, *P* = 0.0257, n = 450).

*Plasmodium falciparum* infection significantly reduced the survivorship of *An. gambiae* that ingested ivermectin (LC_25_) on DPI 14 compared to control mosquitoes that received a primary blood meal without *P. falciparum* (*χ*^2^ = 4.97, *P* = 0.0257, n = 450) (Figure 5). Preliminary data did not indicate that *P. falciparum* reduced the survivorship of *An. gambiae* that co-ingested ivermectin (LC_35_) on DPI 0 compared to control mosquitoes that received only a blood meal (*χ*^2^ = 0.1054, *P* = 0.7454, n = 61), therefore, further replicates were not performed. Preliminary data indicated that *P. falciparum* infection compared to a control blood meal on DPI 0 did not reduce *An. gambiae* survivorship (*χ*^2^ = 0.3518, *P* = 0.5531, n = 59).

## Discussion

This study demonstrates that, in several different combinations, ivermectin is sporontocidal and reduces the prevalence or proportion of *An. gambiae* ingesting an infective *P. falciparum* blood meal that become sporozoite positive. *Plasmodium falciparum*-infectious *An. gambiae* may be more susceptible to ivermectin compared to non-infected mosquitoes. Ivermectin does not appear to lengthen the extrinsic incubation period of *P. falciparum* in *An. gambiae*.

Ivermectin (LC_25_) ingested concomitantly with parasites (DPI 0) had the most dramatic effect on *P. falciparum* sporogony of any ivermectin concentration or DPI combination (Figures 1– 4). While the proportion of mosquitoes that developed oocysts were reduced by ivermectin treatment, the number of oocysts per infected mosquito was not reduced, which implies that ivermectin exerts an ‘all-or-nothing’ effect on oocyst establishment and development. There was a significant reduction in parasite development when ivermectin (LC_25_) was ingested at DPI 6 and DPI 9 but not at DPI 3 (Figure 2). This suggests that ivermectin can affect late-stage but not early-stage oocyst development. Finally, ivermectin (LC_5_) ingested on DPI 0 had no effect on oocyst or sporozoite prevalence (Figure 3), however when ivermectin (LC_5_) was ingested prior to parasites (DPI −3) there was a significant reduction in sporozoite prevalence (Figure 4). These results suggest that ivermectin has a modulating effect on the mosquito host environment that is unfavourable for sporogonic development.

While this report does not attempt to elucidate the mode of action of ivermectin on *P. falciparum* sporogony, the data presented may offer some insight. Ivermectin is a lipophilic molecule that embeds itself in membranes and interacts with transmembrane portions of ion channels [24]. In invertebrates, ivermectin interacts with glutamate-gated chloride ion channels in neuronal and neuromuscular tissues, which leads to flaccid paralysis [9,10,25]. There may also be some interaction of ivermectin with γ-aminobutyric acid-gated chloride channels [26-28]. Neither of these chloride ion channels are present in *P. falciparum* and ivermectin does not have relevant activity against blood-stage *P. falciparum* at concentrations found in humans post oral treatment. Growth of blood-stage *P. falciparum* (K1 isolate) was inhibited by ivermectin but the mean IC_50_ and IC_90_ values were 8.0 and 35.0 μg/ml [29] which are several hundred to thousand times higher than concentrations used in the current study (6.1 and 10.7 ng/ml) and normal levels found in human venous plasma after ingesting ivermectin (150 μg/kg) [30]. Indeed, oral ivermectin (200 μg/kg) treatment of *P. falciparum*-infected humans had no effect on blood-stage parasites [31]. Similarly, no relevant effects of ivermectin were demonstrated against the Apicomplexa coccidian *Eimeria muris* or dozens of species of bacteria and fungi [32]. Nevertheless, it may be possible that unknown ivermectin targets specific to sporogonic *P. falciparum* stages exist which may explain the activity against late-stage but not early-stage oocysts (Figure 2). The broad activity of ivermectin is evident from recent *in vitro* evidence that demonstrates that ivermectin can effect viral replication of human immunodeficiency virus and several arthropod-transmitted Flaviviruses by targeting virus NS3 helicase activity [33] or inhibiting nuclear import [34].

The observed ‘all-or-nothing’ effect of ivermectin on oocyst development in this report is more congruent with a sporontocidal mechanism whereby ivermectin acts on the mosquito rather than the parasite. Ivermectin may alter some aspect of the mosquito midgut physiology that impacts parasite development or the drug may stimulate enhanced anti-*Plasmodium* innate immunity. Regarding the latter possibility, no melanized oocysts from dissected midguts were observed from any control or ivermectin-treated mosquitoes with any ivermectin concentration or DPI combination. The data showing that ivermectin (LC_5_) only inhibits sporogony when ingested DPI −3, but not at DPI 0, suggests that this treatment may alter some aspect of the midgut physiology that prevents parasite establishment, such as mosquito peritrophic matrix formation or blood meal digestion prior to parasite ingestion. Previous work has shown ivermectin to delay the formation and reduce the thickness of the peritrophic matrix in *Aedes aegypti*[35]. Delays in blood meal digestion by *An. gambiae* that had ingested an ivermectin-containing blood meal have been reported previously [4] and were observed in these experiments as well. There is a complex interaction of the *P. falciparum* ookinete with the peritrophic matrix [36,37] and the midgut epithelium [38], which may be affected by ivermectin.

Previous investigators have found accelerated development kinetics of wild strains of *P. falciparum* in wild *Anopheles* in that most ookinetes were formed in the blood meal before twenty-four hours [39], while with the *in vitro* NF54 strain in colonized *Anopheles* the ookinetes were formed in the blood meal after twenty-four hours [40-42]. The ivermectin studies presented here should be replicated with wild *P. falciparum* isolates and various *An. gambiae* strains and other *Anopheles* species to ensure that the effects of ivermectin observed with NF54 represent what occurs in the field. The effects of ivermectin on other *Plasmodium* species in various *Anopheles* species should be investigated as well.

Ivermectin did not appear to lengthen the extrinsic incubation period (*n*), for any ivermectin concentration or DPI combination. More frequent dissections and sporozoite observation on DDPIs 10, 11, 13 and increased replication would provide more data for the general linear mixed models which may be better than the inputs used for this study. Extending dissections beyond DDPI 14 may not provide relevant information as the proportion of infected control mosquitoes at DDPI 7 matched the proportion of infectious control mosquitoes at DDPI 14 (*χ*^2^ = 0.2803, *P* = 0.6717, n = 135) and the proportion of infected ivermectin-treated mosquitoes at DDPI 7 matched the proportion of infectious ivermectin-treated mosquitoes at DDPI 14 (*χ*^2^ = 0.0, *P* = 1, n = 90) (Figure 1). Alternative methods such as enumerating ruptured oocysts at time points beyond DDPI 7 may be necessary to determine if the extrinsic incubation is altered.

A significant, although modest, reduction in *P. falciparum*-infectious *An. gambiae* survivorship compared to uninfected counterparts was demonstrated (*χ*^2^ = 4.97, *P* = 0.0257) (Figure 5). As the effect of *P. falciparum* on *Anopheles* survivorship is debatable [43], further experiments conducted with wild *P. falciparum* isolates combined with enumeration of mosquito mortality prior to ivermectin ingestion are necessary. A reduced susceptibility of *P. falciparum*-infectious *An. gambiae* to ivermectin combined with the fact that *Plasmodium* sporozoite salivary gland infection alters mosquito behaviour would enhance the efficacy of ivermectin MDA for *Plasmodium* transmission control. *Plasmodium*-infectious vectors in the laboratory [44,45] and the field [46,47] have increased mosquito host probing and blood feed more frequently compared to uninfected mosquitoes. In Tanzania, wild *P. falciparum*-infectious *An. gambiae* s.l. were more likely to feed on multiple people per night and feed to repletion compared to uninfected counterparts [47]. Multiple ivermectin-containing blood meals were found to compound *An. gambiae* s.s. mortality at a greater rate than a single ivermectin-containing blood meal [4]. As roughly 80% of people in a village are treated with ivermectin during routine MDAs, it follows that the more frequent host feeding of *P. falciparum*-infectious *An. gambiae* s.s. means that they are more likely to imbibe a blood meal that contains ivermectin or multiple blood meals that contain ivermectin. These findings may further explain some of the reduction in the proportion of *P. falciparum*-infectious *An. gambiae* s.s. post-ivermectin MDA [6], especially if *P. falciparum*-infectious *An. gambiae* are more likely to die than their uninfected counterparts.

Currently, it is not known how much ivermectin is imbibed by a mosquito during blood feeding on an orally treated human. Human pharmacokinetic data for oral ivermectin treatment is based on venous blood samples [30], however ivermectin is found in fat and dermal tissue at two to three times greater concentrations than venous plasma [48]. As mosquitoes blood feed from subdermal capillaries, the amount of ivermectin imbibed may differ from the amount present in venous plasma. This makes it difficult to predict how long laboratory-demonstrated mosquito sub-lethal sporontocidal effect of ivermectin may impact *P. falciparum* transmission dynamics in the field following ivermectin MDA. Concurrent studies are attempting to quantify the concentration of ivermectin imbibed by a mosquito compared to venous and capillary blood concentrations.

This study clearly demonstrates that ivermectin can impact *P. falciparum* sporogony in *An. gambiae* which is a critical factor to reduce *Plasmodium* transmission via ivermectin MDA. Since ivermectin MDA has only been demonstrated to reduce vector transmission, it may be necessary to combine ivermectin MDA with anti-malarial drug treatment to clear parasites from the human population. One disadvantage of mass screening and treatment (MSAT) with anti-malarial drugs is that parasites may be transmitted between the screening and treatment interval [49]. Ivermectin MDA may address this MSAT shortcoming if ivermectin is delivered at the point of sample collection to limit *Plasmodium* transmission between sample processing and anti-malarial drug treatment, however, the safety and efficacy of the co-administration of ivermectin and anti-malarial drugs remains to be validated. *Plasmodium falciparum* gametocyte circulation time has been shown to be reduced post artemisinin-based combination therapy (ACT) [50], however, field evidence demonstrates that *P. falciparum* transmission still occurs post-ACT treatment [51-54]. Primaquine is effective against late-stage gametocytes [55,56] and has been co-administered with ACT in an effort to reduce *Plasmodium* transmission. However, primaquine causes haemolysis in humans with glucose-6-phosphate dehydrogenase deficiency [57,58] which could reduce human compliance with the treatment regimen. As ivermectin has sporontocidal, mosquito lethal, and mosquito sub-lethal effects that prevent *Plasmodium* transmission, and does not cause haemolysis, it may be an ideal agent to combine with ACT.

## Conclusions

Ivermectin is sporontocidal to *P. falciparum* at concentrations sub-lethal to *An. gambiae* and that are present in humans after oral treatment. Mosquito ingestion of ivermectin inhibited sporogony in *An. gambiae* when ingested before, with, or after parasite ingestion. *Plasmodium falciparum*-infectious *An. gambiae* may be slightly more susceptible to ivermectin than uninfected counterparts. As these studies were conducted with cultured *P. falciparum* (NF54) and colonized *An. gambiae* s.s. (G3 strain) it will be necessary to validate results with wild *P. falciparum* isolates and wild-caught *An. gambiae* or with other *Plasmodium* and *Anopheles* species combinations. These results further substantiate the utility of ivermectin MDA to reduce *P. falciparum* transmission.

## Abbreviations

RBC: Red Blood Cell; DMSO: Dimethyl Sulfoxide; DPI: Days Post-Infection; DDPI: Dissected At Day Post-Infection; MDA: Mass Drug Administration; MSAT: Mass Screening And Treatment; ACT: Artemisnin-based Combination Therapy.

## Competing interests

The authors declare that they have no competing interests.

## Authors’ contributions

KCK, BDF, and JHR designed research, KCK performed research, KCK analysed data, KCK, BDF, and JHR wrote and edited the paper, JHR provided reagents. All authors read and approved the final manuscript.
